# Investigating the Role of Resilience, Foreign Language Teaching Enjoyment, and Mindfulness in Predicting Loving Pedagogy in English Language Teaching

**DOI:** 10.11621/pir.2025.0305

**Published:** 2025-09-30

**Authors:** Shokouh Alipour, Saeed Ghaniabadi, Mostafa Azari Noughabi

**Affiliations:** a Shahrekord University, Iran; b Hakim Sabzevari University, Iran

**Keywords:** loving pedagogy, foreign language teaching enjoyment, resilience, mindfulness, EFL teachers

## Abstract

**Background:**

Loving pedagogy is a new concept in the field of applied linguistics and its correlates have not been fully researched.

**Objective:**

To investigate the relations of English as a foreign language EFL) teachers’ resilience, mindfulness, and foreign language teaching enjoyment FLTE) in prediction of their attitudes toward loving pedagogy in Iran.

**Design:**

Iranian EFL teachers N = 255) completed four questionnaires online on Google Forms. In order to analyze the data, standard multiple regression analysis was performed using SPSS version 25.

**Results:**

The results of standardized multiple regression revealed that EFL teachers’ FLTE, resilience, and mindfulness were significant predictors of their loving pedagogy. FLTE was identified as the strongest predictor.

**Conclusion:**

The findings of this study signified the importance of positive psychological factors in creating a pedagogy of love where EFL teachers show kindness and sympathy toward their learners.

## Introduction

Advances in studying positive psychology have led to deeper understanding of positive personality components, psychological traits, and institutional traits Enferad et al., 2025; Wang et al., 2021). Positive psychology offers enhanced understanding into the factors that lead to well-being and success within educational settings Derakhshan et al., 2025; Shoshani et al., 2016; Zou et al., 2025). Focusing on the emotional aspect of learning, positive psychology underscores the influence emotions have within the language teaching environment, emphasizing their central role in effective language acquisition Chen et al., 2024; Dewaele et al., 2019; Wang et al., 2021). Given the recent emphasis on emotions and internal psychological motivations in positive psychology within second-language acquisition SLA) Dewaele & Li, 2020), the idea of loving pedagogy has been examined. Loving pedagogy is defined by Zhao and Li 2021) as “the care, sensitivity, and empathy that teachers have toward their students’ needs, learning experiences, and development” p. 2). The cultivation of positive emotions significantly contributes to students’ development, while loving pedagogy plays a pivotal role across various domains of EFL learning and teaching Barcelos, 2020).

The pedagogy of love is an important part of education, and teachers who truly care for their students typically perform better Yin, Loreman, et al., 2019). In the domain of language teaching, there is not much research on loving pedagogy, but if the antecedents to loving pedagogy are included, this is tantamount to one single study Zhi & Wang, 2023). The three most important predictors of the pedagogy of love are mindfulness, enjoyment of teaching, and resilience. Mindfulness is a process whereby teachers can control their own emotions in order to attend to the present moment and not be distracted by worries about the past or future Li, 2021). Mindful teachers are more prepared to develop resilience in their working lives, where they may have to use resilience to be able to keep going when they encounter occupational challenges, and because of this, substantial evidence shows there is a correlation between mindfulness, resilience, and educators’ well-being, job satisfaction, and professional engagement Skinner & Beers, 2016). Research has considered mindfulness as a way to diminish stress and increase the psychological well-being of teachers and sustain active engagement Moyano et al., 2023; Ramasubramanian, 2017), and a research area termed “mindful pedagogy” is emerging. Mindfulness contributes toward loving-kindness through the reduction of aversion toward caring behaviors, enabling awareness and empathy to be cultivated. This allows educators to engage in a loving pedagogy that seeks to develop positive emotional attachment and supportive learning environments, and teachers who engage in this form of pedagogy may contribute to student engagement and well-being Zolfaghari et al., 2024).

Resilience is considered a stable characteristic by some researchers, others see it as an evolving element Chiccetti, 2010; Hiver, 2018). Resilience has an important function in successful stress management, adaptativity, and emotional control X. Wang et al., 2024). The Foreign Language Teaching Enjoyment FLTE) construct of Ergün and Dewaele 2021) consider it as parallel to the enjoyment experienced by language learners. FLTE has three sub-areas: personal enjoyment in the classroom, social enjoyment, and appreciation of the learners. FLTE can play an important role in a loving pedagogy that characterizes teachers’ interest in the profession by caring and positive emotions.

Resilience and mindfulness are significant factors in promoting a loving pedagogy within the field of foreign language teaching. Resilience allows teachers to adapt and recover from challenges and stress, making a suitable learning environment for their students Mansfield et al., 2016). Additionally, mindfulness enhances empathy and understanding of students’ needs, enabling teachers to create a more inclusive and engaging classroom experience Kabat-Zinn, 2009). By incorporating resilience and mindfulness into their teaching practice, English language teachers can foster a loving pedagogy that emphasizes emotional connections, empathy, and well-being. The present study seeks to examine the ways in which loving pedagogy can be cultivated through the combined contributions of resilience, foreign language teaching enjoyment, and mindfulness. By exploring the interplay of these factors, the research aims to shed light on how they collectively foster an environment of loving pedagogy and enrich the overall teaching/learning experience.

This research applies an innovative methodology to the study of the interaction of mindfulness, enjoyment of teaching foreign languages, and resilience in the framework of loving pedagogy. This study is one of the first to research these factors with the use of theories of loving pedagogy and positive psychology, and offers new information about how these factors can be combined in order to enhance the process of learning. The research provides usable results for instructors wishing to foster a more loving learning environment. Furthermore, the findings promise to energize future studies, enriching our understanding of loving pedagogy and its interwoven components. In so doing, it offers a solid foundation for further research and innovation within this area of study.

## Literature Review

### Loving Pedagogy

The notion of love within the educational context exerts a profound impact on the comprehensive development of learners, notably in regard to their social and emotional growth Yin, Loreman, et al., 2019). The term loving pedagogy is delineated as a non-romantic form of intimacy, distinguished by reciprocal trust, respect, and care between educators and their pupils Ghiasvand & Sharifpour, 2024). This approach is fundamentally anchored in the assumption of positive psychology, which addresses the emotional aspects of language instruction that have frequently been neglected due to cultural sensitivities Wang et al., 2022).

It is clear that teaching is not just transferring knowledge and getting academic outcomes; having a loving relationship and intimacy between teachers and students is a fundamental part of schooling. Loreman 2011) articulates the concept of love within the educational sphere as comprising different elements: kindness, sacrifice, empathy, forgiveness, intimacy, bonding, acceptance, and community. These are fundamental to fostering effective learning environments. According to these elements, the dispositions towards loving pedagogy DTLP) scale was designed Yin, Loreman, et al., 2019).

Although some studies have been conducted in the pedagogy of love, few were in non-Western areas. In the field of emotional and psychological factors, some studies have been done. For example, empirical evidence suggests that inclinations towards loving pedagogy can markedly alleviate teacher burnout by bolstering self-efficacy. In the realm of language education, loving pedagogy encompasses positive and compassionate dispositions of educators towards students, thereby mitigating burnout while promoting well-being and effectiveness within the classroom environment Chen, 2023). Another study involving a cohort of Chinese EFL educators showed a positive correlation between loving pedagogy and the psychological well-being and work engagement of teachers Li & Miao, 2022). Loving pedagogy cultivates a nurturing classroom atmosphere, which is essential for fostering student engagement and enhancing academic performance Zhao & Li, 2021). The emotional support facilitated through loving pedagogy has the potential to enhance student confidence and participation in the language acquisition process Ghiasvand & Sharifpour, 2024). The study of Liu and Fan 2025) shows that language teachers’ support has a significantly direct impact on learners’ willingness to communicate. A few studies have been conducted on the relationships between loving pedagogy and various emotional factors, including resilience, mindfulness, and foreign language teaching enjoyment.

### Teacher Resilience

Resilience is considered as an essential attribute of educators Hiver, 2018) and has become an international concern in language learning and teaching Chu et al., 2024). While a segment of academia has posited resilience as a stable characteristic, a contrasting viewpoint perceives resilience as an evolving phenomenon Cicchetti, 2010; Hiver, 2018). Regardless of the current discourse surrounding the precise definition of teacher resilience, a burgeoning consensus exists that this complicated concept signifies educators’ positive transformation to challenging circumstances Gu, 2014). Teacher resilience has been linked with different psychological factors. As articulated by Day 2014), teacher resilience correlates with being engaged with the job. Furthermore, extant literature indicates that educators possessing great degrees of resilience are more inclined to transition from misery to pleasure. Research has shown close links between teachers’ resilience, enthusiasm, motivation, and self-efficacy Beltman et al., 2011; Peters & Pearce, 2012). Teacher resilience can also enhance educators’ progression toward enhanced job commitment Mansfield et al, 2016).

Teacher resilience is a foundational element that bolsters language teacher immunity to professional hurdles Hiver, 2017). In another study, multiple regression was used to analyze positive correlations of Iranian EFL educators’ reflective teaching practices and their resilience Ayoobiyan & Rashidi, 2021). Resilient and “gritty” language educators exert considerable effort to attain efficient working conditions despite adversities and challenges Hiver, 2018; Sudina, Vernon, et al., 2021).

A teacher with higher resilience a has higher level of devotion Yin, Huang, et al., 2019). Promoting engagement and resilience among EFL teachers contributes to the development of L2 grit, leading to more efficient teaching practices and positive student learning outcomes. The interplay of resilience, well-being, and L2 grit significantly contributes to foreign language teaching enjoyment FLTE). These positive psychological factors enhance teachers’ experiences and effectiveness in the classroom Derakhshan et al., 2022).

### Teacher Mindfulness

Mindfulness is a mediator in reducing teacher burnout, including EFL educators, by enhancing psychological capital and promoting resilience Liu & Du, 2024). Mindfulness enhances EFL teachers’ emotional control, which in turn boosts their commitment to teaching Wang, 2023). Teachers with higher mindfulness report more optimism and commitment, contributing undoubtedly to students’ academic success Lu, 2021). The ability to navigate challenges effectively allows teachers to transform adversities into opportunities for professional development Miri et al., 2015).

Mindfulness can be examined through the lens of positive psychology, conceptualizing it as a trait shaped by both inherent and learned factors Duan, 2014). People exhibiting higher mindfulness are more likely to be open-minded and cognizant of the present, characterized by heightened awareness and attention Kabat-Zinn, 1994). In the medical domain, it has been proved that mindfulness can enhance positive emotions and decrease negative ones. Subsequently, as the inquiry into mindfulness broadened beyond clinical settings, scholars identified its capacity to improve metacognition Garland et al., 2009), promote individual well-being Brown & Ryan, 2003), and moderate negative emotional states Brown & Ryan, 2003; Coffey et al., 2010).

By studies on positive psychology and its integration into educational contexts, researchers have increasingly focused on the influence of mindfulness on academic emotions such as enjoyment, pride, anxiety, and boredom Senker et al., 2021; Xie & Guo, 2023). Despite the extensive research on the connections between mindfulness and emotions, as well as the relationship between social support and emotions in academic contexts, there is a notable lack of studies examining the link between mindfulness, resilience, loving pedagogy, and foreign language teaching enjoyment, indicating a need for further empirical exploration.

### Foreign Language Teaching Enjoyment FLTE)

Ergün and Dewaele 2021) proposed the concept of foreign language teaching enjoyment FLTE) as a counterpart to learners’ enjoyment in foreign language acquisition. This concept encompasses three key components: personal enjoyment, social enjoyment within the classroom, and the gratitude expressed by learners. To assess FLTE, they created a 9-item instrument and discovered a positive correlation between the resilience and well-being of second/foreign language L2) teachers and FLTE, with resilience emerging as the most significant predictor. However, they emphasized the need for further research to identify individual teacher characteristics that could enhance FLTE. Teachers who maintain a positive mental state and possess coping strategies can enjoy teaching more Wei et al., 2024).

Fathi et al. 2024) discovered that the professional identity of EFL teachers impacted their involvement through determined actions and pleasure in teaching. Mierzwa 2019) did a mixed-method study to explore FLTE among teachers, which found that Polish teachers reported high degrees of FLTE. Mierzwa also noted that factors such as teachers’ gender, years of experience, and type of school did not significantly affect their FLTE, apart from any psychological variables. King et al. 2020) underscored the crucial role of emotions for language instructors, suggesting that the regulation of these emotions is influenced by personality traits or training, which may vary significantly among educators. Investigating the foundational dimensions of enjoyment within foreign language classrooms, Jin and Zhang 2021) found that the enjoyment derived from student support, in conjunction with the enjoyment stemming from teacher support, exerted a positive influence on foreign language learners’ enjoyment of English language acquisition, which in turn served as a predictor of their linguistic proficiency. Jiang and Dewaele 2019) emphasized the significant impact of teacher-related factors on the enjoyment experienced by language learners in foreign language acquisition. Emotion regulation was investigated as a factor that impacts foreign language teaching enjoyment Azari Noughabi et al., 2022).

The resilience, well-being, and L2 grit of EFL teachers have a substantial effect on their FLTE. Furthermore, among Iranian EFL educators, teacher L2 grit emerged as the most significant predictor of FLTE. This indicates that teachers who exhibit grit, resilience, and a positive outlook are more inclined to find satisfaction in their classroom teaching experiences Derakhshan et al., 2022). Another study discovered that mindfulness positively affects teacher engagement and reduces burnout and that FLTE partially mediates the relationship between mindfulness and teacher engagement Yang et al., 2023). Recently in a cross-cultural study in Iran and Turkey, it was discovered that factors influencing foreign language teaching enjoyment include personal feelings, enhancing factors, challenges faced, individual coping strategies; this study also suggested solutions for improvement Azari Noughabi et al., 2024).

Up to now, there have not been any studies of the relationship between FLTE and loving pedagogy. Loving pedagogy is rooted in positive psychology, focusing on emotions as critical components of effective language education Wang et al., 2022). EFL teachers who embrace loving pedagogy and are open to adversities demonstrate resilience and adaptability in their teaching. It not only enhances their pedagogical effectiveness, but also fosters a positive learning environment for students. Positive psychology facilitates the development of emotional resilience and the establishment of social connections, both of which are indispensable for the successful acquisition of language. Positive psychology promotes emotional, social, and psychological wellbeing in language education, and enhances new ways to think about language learning Mercer, 2017). Higher levels of loving pedagogy correlate with improved teacher well-being, which can create a better learning environment for language learners Chen, 2023). Mindfulness positively affects teacher engagement and reduces burnout, and FLTE partially mediates the relationship between mindfulness and teacher engagement Yang et al., 2023). The positive language education paradigm merges the processes of language acquisition with personal growth, with the objective of fostering an integrative educational experience MacIntyre, 2021).

Despite the abundance of research exploring the field of loving pedagogy in various educational contexts, there is a conspicuous dearth of studies examining the interplay of mindfulness, FLTE, resilience, and loving pedagogy within non-Western settings, thereby underscoring the need for further investigation into these less-explored, yet potentially significant aspects of educational practice. The present study aims to answer the following research question:

- To what extent can Iranian EFL teachers’ loving pedagogy be predicted by their mindfulness, FLTE, and resilience?

## Methods

### Participants

A snowball sampling technique was utilized to recruit 255 Iranian EFL teachers, who were contacted online mostly via Telegram and Gmail) from different universities and institutes in Iran, to participate in an online survey. Out of the total participants, 214 were female and 41 were male, with an average age of 30.82 years *SD* = 8.33). The average teaching experience among participants was 8.03 years, with a standard deviation of 6.29 years. Their teaching experience ranged from 1 to 40 years. Most of the participants had majored in English language teaching 61.2%). The majority of the participants 48.2 %) had a Master’s degree and 31% of them had a Bachelor’s degree. The majority of participants were teachers in private language institutes 63.1%), followed by secondary and high school 15.3%). Consent forms were obtained from all the participants, and the participants were ensured that their confidentiality would be maintained. Demographic information of the participants is shown in *Table 1*.

**Table 1:** Demographic Information of the Participants

Variable	Frequency *N*)
Major	English Language Teaching = 156 English Language Translation = 42 English Language Literature = 29 Other = 28
Work Context	Secondary/High School = 39 Institute = 161 College/University = 29 Other = 26
Academic Degree	Bachelor of Arts = 79 Master of Arts = 123 Ph.D. = 19 Other = 34

All participants were Iranian. Iranian EFL teachers typically face a series of work-related challenges such as heavy workload, students’ low motivation, and overcrowded classes Azari Noughabi et al., 2024). Yet, they show high levels of FLTE, which indicates that they like their job despite its typical difficulties.

### Instruments

#### Teacher Resilience Scale

The 16-item Language Teacher Resilience Scale, developed by Liu and Chu 2022) was employed. It includes three subscales: tenacity *e.g.*, “I can achieve my goals, even in the face of English teaching challenges”), optimism *e.g.*, “I am able to adapt to change”), and coping style *e.g.*, “I prefer to take the lead in English teaching problem solving”). Participants rated items on a 5-point Likert scale, with responses ranging from “strongly disagree” 1) to “strongly agree” 5). Previous research has provided evidence supporting the reliability and validity of this scale Liu & Chu, 2022). In the present study, the internal consistency of the Resilience Scale, assessed using Cronbach’s alpha, was .79.

#### The Foreign Language Teaching Enjoyment Scale

The Foreign Language Teaching Enjoyment Scale was employed Ergün & Dewaele, 2021). This scale has been adapted from the short form of students’ foreign language enjoyment scale Botes et al., 2021). This 9-item scale assesses FLTE through three factors: personal enjoyment (“I enjoy it”), student appreciation in the foreign language classroom (“The students in the EFL class are stimulating”), and social enjoyment (“We form a tight group in the EFL class”). Participants rated items on a 5-point Likert scale, with responses ranging from “strongly disagree” to “strongly agree”. Previous research supported the reliability and validity of this scale Derakhshan et al., 2022). The internal consistency of this scale in the present study was acceptable α = .839).

#### Mindfulness in Teaching Scale

The Mindfulness in Teaching Scale, developed and validated by Frank et al. 2016), comprises two dimensions: intrapersonal mindfulness *e.g.*, “When I am teaching, I find myself doing things without paying attention”) and interpersonal mindfulness *e.g.*, “I am aware of how my moods affect the way I treat my students”). The scale consists of 14 items rated on a 5-point Likert scale, with responses ranging from “strongly disagree” to “strongly agree.”. Previous research provided evidence that this scale is a reliable and valid measure of teacher mindfulness. In the current study, the internal consistency of this scale was satisfactory α = .752).

#### Dispositions Toward Loving Pedagogy Scale

The Disposition Toward Loving Pedagogy Scale which developed by Yin, Loreman, et al. 2019). It is a 29-item survey which measures six elements of a loving pedagogical approach, including acceptance of diversity *e.g.*, “I engage in classroom activities specifically aimed at encouraging acceptance of diversity in students”), intimacy *e.g.*, “I encourage students to ask for and provide forgiveness”), bonding and sacrifice *e.g.*, “I engage in an active student-teacher learning partnership by working directly with individual students”), empathy *e.g.*, “I deliberately engage in weekly kind acts with my students in the context of my teaching”), forgiveness *e.g.*, “A student who asks for forgiveness should be granted forgiveness, no matter what he or she has done”), and kindness in the pedagogical context *e.g.*, “Being kind to students in important to me”). Each item in the scale was rated on a 4-point Likert scale ranging from 1 strongly disagree) to 4 strongly agree). The reliability of this scale in the present study was acceptable α = .904).

### Data Collection

In order to collect data, four scales were created using Google Forms to measure EFL teachers’ resilience, loving pedagogy, FLTE, mindfulness. These survey forms were designed to be user-friendly and easy to access, allowing for a large set of data to be collected from a diverse group of English language teachers. The survey items were in English and no deadline was set to return the forms. The process of data collection took about two months, from July 2024 to August 2024. All the participants had access to researchers to ask clarifications for items. Then, 255 forms were completed.

### Data Analysis

After the survey forms were completed, the data was screened for missing values. No missing data was found among the completed questionnaires, as all items were designed in a way that required respondents to select an answer for each item. The dataset was checked to make sure that there were no outliers. Next, standard multiple regression analysis was performed using SPSS version 25 to examine the extent to which the predictive variables resilience, mindfulness, and foreign language teaching enjoyment) could explain the variance in the dependent variable loving pedagogy).

## Results

After ensuring the adequacy of the sample size based on the criteria presented by Tabachnick and Fidell 2013), *N* > 50 + 8 *m*, *m* = number of independent variables, the normality of data was checked and data screening was done. The results of the descriptive statistics, shown in *Table 2*, indicates that the data was normally distributed, as the skewness and kurtosis indices were in an acceptable range of ±2 Kunnan, 1998).

**Table 2:** Descriptive Statistics

	M	SD	Skewness		Kurtosis	
			Statistic	Std. Error	Statistic	Std. Error
Resilience	3.999	.426	–.115	.153	–.571	.304
Mindfulness	2.773	.447	.226	.153	–.081	.304
Loving Pedagogy	3.249	.415	–.348	.153	–.401	.304
Teaching Enjoyment	4.003	.586	–.262	.153	–.373	.304

Multivariate normality was checked via the Cook and Mahalanobis distance values. The estimated Mahalanobis value 12.325) and Cook value (.037) were in the acceptable range provided by Larson-Hall 2010) Mahalanobis value < 15 and Cook value < 1). The tolerance values were also checked to ensure lack of multicollinearity tolerance value > .10). The estimated tolerance values (.152, .193, .299) fell within the acceptable range Tabachnick & Fidell, 2013). Additionally, we measured Variance Inflation Factor VIF) values to check the collinearity of the data. The estimated VIF values 1.520, 1.524, and 1.005) were acceptable based on the criteria suggested by Larson-Hall 2010, VIF < 5).

Pearson correlations analysis was conducted to check the linearity of the relationships among the variables. As *Table 3* indicates, there were significant and linear relationships between the predicting variables resilience, mindfulness, and FLTE) and the independent variable loving pedagogy).

**Table 3:** Correlations

	Loving Pedagogy	Teaching Enjoyment	Resilience	Mindfulness
Loving Pedagogy	1.00			
Teaching Enjoyment	.501^**^	1.00		
Resilience	.443^**^	.585^**^	1.00	
Mindfulness	.120^*^	.142^*^	.167^*^	1.00

Note. ^*^ p < .05, ^**^ p < .01

Next, standard multiple regression was conducted to analyze the unique predictive power of EFL teachers’ resilience, mindfulness, and FLTE in explaining their tendency to follow loving pedagogy. The model summary, in *Table 4*, showed that EFL teachers’ resilience, mindfulness, and FLTE explained 30% of the variance of their dispositions toward loving pedagogy.

**Table 4:** Model Summary Results

R	R Square	Adjusted R Square	Std. Error of the Estimate	Change Statistics				
				R Square Change	F Change	df1	df2	Sig. F Change
.555	.308	.300	.347	.308	37.325	3	251	.000

*Note. Dependent variable: Loving pedagogy; Predicting variables: Mindfulness, Resilience, and FLTE.*

Moreover, the results of ANOVA, shown in *Table 5*, proved that the model of the predictors of loving pedagogy was significant; *F*3, 251) = 37.325, *R*^2^ = .308, *p* < .001. Accordingly, the results revealed that EFL teachers’ loving pedagogy was significantly predicted by their FLTE, resilience, and mindfulness.

**Table 5:** ANOVA Results

	Model	Sum of Squares	df	Mean Square	F	Sig.
1	Regression	13.511	3	4.504	37.325	.000
	Residual	30.286	251	.121		
	Total	43.798	254			

In particular, as indicated in *Table 6*, it was revealed that EFL teachers’ FLTE was a stronger predictor *B* = 5.693, *t* = 11.17, *p* < .001) of their loving pedagogy, compared to their resilience *B* = .238, *t* = 3.672, *p* < .001), and mindfulness *B* = .152, *t* = 2.889, *p* < .01).

**Table 6:** Coefficients

	Standardized Coefficients					95.0% Confidence Interval for B	
	B	Coefficients Std Error	Beta	t	Sig.	Lower Bound	Upper Bound
Mindfulness	.141	.049	.152	2.889	.004	.045	.237
Resilience	.232	.063	.238	3.672	.000	.108	.356
Teaching enjoyment	.261	.046	.368	5.693	.000	.171	.351

In sum, the findings of the present study showed that EFL teachers with higher levels of FLTE, resilience, and mindfulness were more likely to incline toward loving pedagogy.

## Discussion

Because of the unique nature of the idea of loving pedagogy, this study aimed to identify its antecedents. The findings showed that EFL teachers’ enjoyment, mindfulness, and resilience were significant predictors of their dispositions toward loving pedagogy. The results support the principles of positive psychology Budzinska & Majchrzak, 2021; MacIntyre, 2021), suggesting that positive emotions *e.g.*, FLTE) and psychological factors *e.g.*, resilience and mindfulness) can lead to fruitful teaching and learning Derakhshan et al., 2022, 2023; Yang et al., 2023). Moreover, the results correspond with the broaden-and-build theory Fredrickson, 2001), indicating that concentrating on positive emotions and elements can result in favorable outcomes. Positive emotions can be said to transfer from one individual to another Moskowitz & Dewaele, 2021).

Having a good rapport with students can lead to a satisfying emotional state in educators. This, in turn, enables teachers to be more sympathetic and embrace a pedagogy of love Yin, Loreman, et al., 2019). The findings further support the application of positive psychology in the field of foreign language learning Mercer, 2017; Wang, et al. 2023). The findings complement the contribution of Zolfaghari et al. 2024) by introducing further predictors of EFL teachers’ tendency toward loving pedagogy.

Regarding the research question, the results of multiple regression analysis indicated that EFL teachers’ enjoyment was a stronger predictor of their tendency toward loving pedagogy than mindfulness and resilience. This finding is partially in line with earlier studies on loving pedagogy such as that by Zolfaghari et al. 2024) where, following Ergün & Dewaele 2021), FLTE is believed to indicate the personal and social enjoyment of EFL teachers alongside the appreciation of learners. Accordingly, educators who receive positive feedback and expressions of appreciation from their students are more likely to navigate challenges effectively and, in turn, endeavor to reciprocate this with appreciation and kindness toward their students. Therefore, it can be clearly justified that a teacher with higher levels of FLTE adopts a more positive approach toward loving pedagogy.

The findings also indicated that resilience was positively correlated with EFL teachers’ mindfulness and loving pedagogy. Research has demonstrated that resilience strongly contributes to FLTE Derakhshan et al., 2022; Ergün & Dewaele, 2021). As enjoyment has been shown by Zolfaghari et al. 2024) as a strong predictor of loving pedagogy, it can be posited that resilience also plays a contributory role in fostering loving pedagogy. Furthermore, numerous studies have established a strong correlation between resilience and engagement *e.g.*, Derakhshan et al., 2023; Shahvarani et al., 2023). The relationship between engagement and loving pedagogy has also been substantiated by Wang et al. 2023), who identified a significant association between these two constructs see also Derakhshan et al., 2023). Therefore, considering that resilience contributes to engagement and that engagement is recognized as a correlate of loving pedagogy, it follows that more resilient educators are more predisposed to embrace loving pedagogy. This inclination arises from their capacity to tolerate differences, challenges, and difficulties within the classroom environment. In discussing loving pedagogy, Yin, Loreman, et al. 2019) stipulate that one of its outcomes is the teachers’ openness to adversities and their heightened tolerance in addressing the challenges presented by the classroom environment.

The present study found a significant correlation between EFL teachers’ mindfulness and loving pedagogy. According to Yang et al. 2023), mindful teachers exhibit higher FLTE in their classrooms and lower burnout. They demonstrate higher well-being and greater engagement in their teaching practices. A mindful teacher sets aside personal challenges and fully commits to their professional responsibilities. Notably, one of the sub-branches of loving pedagogy, as identified by Yin, Loreman, et al. 2019), is sacrifice; thus, a mindful teacher practically dedicates themselves to their job. Specifically, when in the classroom, they entirely disconnect from external distractions and engage with their work at a high level of professional commitment.

Given that engagement and FLTE have been shown to be significantly associated with loving pedagogy, it can be inferred that mindful teachers are more inclined toward loving pedagogy due to their professional commitment, enjoyment of teaching, and willingness to sacrifice for the benefit of their students. In sum, the findings indicate that mindful, resilient EFL teachers are more likely to follow a pedagogy of love where EFL teachers tolerate adversities, consider the learners’ individual differences, show kindness in the class, devote themselves to their careers, and create a caring atmosphere for their learners Zolfaghari et al., 2024).

## Conclusion and Implications

The results suggest that resilience, mindfulness, and foreign language teaching enjoyment are important predictors of loving pedagogy in English language teaching. These results highlight the significance of fostering these characteristics in teachers in order to establish a positive and nurturing learning environment for students. The results suggest that teachers can benefit from mindfulness-based interventions, such as meditation, yoga, or other mindfulness practices, to develop their resilience and promote a more loving pedagogy in the classroom. Professional development programs for teachers can focus on strategies for improving resilience, mindfulness, and foreign language teaching enjoyment, such as stress management techniques, mindful communication strategies, and self-care practices.

Education policymakers ought to think about integrating resilience and mindfulness training into teacher training programs to assist educators in managing stress and overcoming challenges in their classrooms. School administrators and leaders can promote a culture of mindfulness and resilience by providing resources and support for teachers to develop these qualities. School administrators can establish a supportive and collaborative environment in which teachers feel comfortable seeking help and support when they are struggling with stress or burnout. Parents and guardians can also play a role in supporting teachers by communicating with them regularly, offering feedback and suggestions, and modeling positive attitudes towards learning and teaching.

Although this study revealed significant findings, it is essential to acknowledge its limitations. First, it did not include qualitative data from interviews or observations, nor did it incorporate open-ended questions. To obtain a deeper understanding of the relationship between resilience, mindfulness, enjoyment in foreign language teaching, and loving pedagogy, future research should utilize mixed-method approaches. Second, this study focused on a group of teachers from Iran. Thus, it is crucial to explore these relationships across diverse educational contexts and among various populations of teachers to assess the generalizability of the findings. Future investigations could include a variety of teaching environments, such as urban versus rural schools, as well as differences in educational systems and cultural contexts. This would help identify whether the observed relationships hold true across different settings. Third, this study did not take teachers’ demographic variables into consideration. Therefore, incorporating a wider range of teacher demographics, including factors such as age, teaching experience, and cultural background, can enhance our understanding of how these variables interact with resilience, mindfulness, and pedagogical enjoyment.
